# Assessment of total (anti)oxidant status in goat kids

**DOI:** 10.5194/aab-64-139-2021

**Published:** 2021-04-26

**Authors:** Stefano Cecchini, Francesco Fazio

**Affiliations:** 1 Department of Sciences, University of Basilicata, Viale dell'Ateneo Lucano 10, 85100 Potenza, Italy; 2 Department of Veterinary Sciences, Polo Universitario Annunziata, University of Messina, 98168 Messina, Italy

## Abstract

The redox potential of goat serum was assessed by different spectrophotometric assays. Among them, three methods are commonly applied for the evaluation of the oxidative (reactive oxygen metabolites, ROMs, and total oxidant status, TOS) and nitrosative (NO${}^{⚫}$ metabolites, ${\mathrm{NO}}_{x}$) stress, and four methods for the evaluation of the antioxidant status: the total antioxidant capacity (TAC) based on the ferric reducing ability of plasma (FRAP), the total antioxidant activity (TAA) based on the reduction of the coloured ABTS${}^{⚫+}$ radical cation, the free radical scavenging activity (FRSA) based on the reduction of the purple DPPH${}^{⚫}$, and the total thiol levels (TTLs) based on their interaction with DTNB to form a highly coloured anion. Besides, myeloperoxidase (MPO) and ceruloplasmin oxidase (CP) activities were also assessed.

Except for TAA, analytical data showed a great inter-individual variation for both oxidant and antioxidant assays. ROMs were strongly correlated with CP, while TOS with MPO and TAC. Furthermore, a tendency between TOS and FRSA was shown. ${\mathrm{NO}}_{x}$ was correlated with TAC and TAA, and a tendency with TOS was shown. No correlations appeared among the antioxidant assays, even if a tendency between TAC and TAA was evidenced, but TAC was correlated with MPO activity. The observed correlation between ROMs and CP is discussed as a possible analytical interference.

The absence of correlation among the antioxidant biomarkers suggests the simultaneous use of a panel of tests to verify any changes in the redox balance, mainly in livestock in which reference values for each biomarker are lacking.

## 1 Introduction

Oxidative stress (OS), generally defined as an imbalance between the production of reactive oxygen and nitrogen species (ROS and RNS) and the capacity to detoxify reactive intermediates or to repair the consequent damage by an appropriate antioxidant defence system, has been associated with several physiological and pathological conditions in livestock (Lykkesfeldt and Svendsen, 2007; Celi, 2011a). In ruminants, a close relationship among OS, chronic diseases, and physiological stage (Celi, 2011a) and among energy balance, metabolism, diet, production traits, and redox status, as a balance between oxidant and antioxidant levels, was evidenced (Celi and Gabai, 2015; Di Trana et al., 2015; Gatellier et al., 2004).

The growing interest in the effect of OS and antioxidant-enriched diets on livestock welfare and the quality of derived products has prompted the use of analytical methods, already in use in human medicine for many years, also for the evaluation of the redox potential in animal biological samples (Cecchini et al., 2019; Celi, 2011a; Chávez-Servín et al., 2018; Di Trana et al., 2015; Giorgio et al., 2019; Todaro et al., 2017).

These assays are primarily intended for use in clinical studies, but their application in experiments is not excluded. Anyway, in contrast to human medicine, the lack of reference values for OS biomarkers in veterinary science makes it difficult to determine whether and when animals are experiencing OS or benefit from antioxidant-enriched diets (Cecchini et al., 2018). However, among the methods applied to assess the oxidant/antioxidant status of biological samples, most of them are spectrophotometric assays and can be automated and applied on clinical auto-analysers, thus allowing rapid and inexpensive data collections.

Generally speaking, these assays are based on the principle that the antioxidant potential of a biological sample is measured as the content of free radicals scavenged by a test solution or the capacity to reduce an oxidized chemical substance, whereas the oxidant potential is measured as the content of substances able to oxidize a chemical compound in a test solution (Alberti et al., 2000; Erel, 2005; Ghiselli et al., 2000). Some of these assays have recently been tested in biological samples of livestock other than serum or plasma, such as saliva in sheep (Rubio et al., 2019) and the exhaled breath condensate in horses (Po et al., 2013). The obtained data produced effective results in the analytical validation, proposing them for a less invasive method of OS evaluation (Rubio et al., 2019; Po et al., 2013).

Although the analytical data obtained with these methods are considered as the cumulative action of all the oxidant/antioxidant substances present in biological samples, thus providing an integrated parameter rather than the simple sum of measurable substances, one of the critical points adduced to their application is that the results obtained with different methodologies are not always comparable, often returning inconsistent and non-straightforward data. This seems to depend on which technology is used for their assessment (Cao and Prior, 1998; Celi, 2011b; Jansen and Ruskovska, 2013; Rubio et al., 2016). Therefore, the comparison of different analytical assays represents the pivotal factor to understand the effective status of the redox potential in biological samples.

Considering the current literature, this study aimed to carry out an extensive comparative analysis of the results obtained with different spectrophotometric methods to measure the oxidant/antioxidant status in goat serum. To avoid unknown influences due to uncontrolled environmental factors, the assays were tested on a group of healthy young specimens in which a reduced level of oxidants is normally observed compared to older animals (Soriano et al., 2015). Alongside the classic methods of evaluation of the redox potential, the activity of myeloperoxidase, a neutrophil enzyme that promotes oxidative stress by using hydrogen peroxide to catalyse the production of strong oxidants during inflammation, and the oxidase activity of ceruloplasmin, as a relevant inhibitor of myeloperoxidase, were also analysed.

## 2 Materials and methods

### 2.1 Animals and blood sampling

Blood samples were obtained from 20 kids of Camosciata delle Alpi goat breed aged three months and an average weight of 15.0±1.0 kg, randomly chosen from a population of 60 female kids and their 50 dams raised on a commercial farm. The kids were bred all together and housed indoors and were naturally suckled and further fed with pasture hay ad libitum and with 300 g d${}^{-1}$ of a commercial concentrate, containing 17 % of crude protein, 3 % fat, 7.30 % crude fibre, and 9.90 % ash. All kids were clinically healthy and free from internal and external parasites. The evaluation of health status was based on rectal temperature, heart rate, respiratory rate, appetite, and faecal consistency (data not shown). Blood samples, collected by a veterinary officer of the Italian National Health System during the compulsory, official eradication and surveillance programmes on brucellosis for which ruminants must be periodically sampled, were drawn from the external jugular vein using vacutainer tubes without anticoagulant. Serum was obtained after clotting by centrifugation (3000 rpm for 10 min at 4 ${}^{\circ }$C) and stored at -80 ${}^{\circ }$C until analyses. All procedures were carried out in strict accordance with the European legislation regarding the protection of animals used for scientific purposes (European Directive 2010/63), as recognized and adopted by the Italian law (DL 2014/26). No animal has suffered as a consequence of blood sampling, as evidenced by the clinical examination.

### 2.2 Analytical methods

Reactive oxygen metabolites (ROMs) were analysed as described by Alberti et al. (2000). The method measures the hydroperoxide levels in a sample, and it is based on the principle that hydroperoxides, in the presence of iron ions released from proteins by an acidic buffered solution, are able to generate alkoxyl (R-O*) and peroxyl (R-OO*) radicals. These radicals oxidize an alkyl-substituted aromatic amine (N,N-diethyl-*para*-phenylenediamine, DEPPD) generating a pink-coloured derivative which is spectrophotometrically measured. Briefly, 10 µL of samples in duplicate was added to wells of a microtitre plate. Subsequently, 200 µL of a solution containing 0.37 mM DEPPD and 2.8 mM iron (II) sulfate heptahydrate in 100 mM acetate buffer, pH 4.8, was added to each well. After incubation (30 min at 37 ${}^{\circ }$C) the optical densities (ODs) were read at 530 nm against a blank using a microplate reader (Model 550, BioRad). The assay was calibrated with *tert*-butyl hydroperoxide (t-BHP), and the results are expressed in terms of t-BHP equivalents (mM).

Total oxidant status (TOS) was analysed as described by Erel (2005). The method is based on the principle that oxidants present in the sample oxidize the ferrous ions, previously bound to the chelator o-dianisidine dihydrochloride, to ferric ions. Subsequently, the ferric ions make a coloured complex with the chromogen xylenol orange in an acidic medium which is spectrophotometrically measured. Briefly, 35 µL of samples in duplicate was added to wells of a microtitre plate and mixed with 225 µL Reagent 1 (xylenol orange 150 µM, NaCl 140 mM and glycerol 1.35 M in 25 mM H${}_{2}$SO${}_{4}$ solution, pH 1.75), and the ODs were read at 550 nm against a blank in the microplate reader. After that, 11 µL Reagent 2 (ferrous ion 5 mM and o-dianisidine 10 mM in 25 mM H${}_{2}$SO${}_{4}$ solution) was added to the mixture. After 5 min at 37 ${}^{\circ }$C, ODs were again read at 550 nm. The assay was calibrated with t-BHP, and the results are expressed in terms of t-BHP equivalents (µM).

Nitric oxide radical (NO${}^{⚫}$) metabolites (${\mathrm{NO}}_{x}$), namely nitrite and nitrate (${\mathrm{NO}}_{x}$), were quantified as described by Miranda et al. (2001) by the acidic Griess reaction after the reduction of nitrate to nitrite by vanadium(III) chloride (VCl${}_{3}$). The Griess reaction is based on a simple colorimetric reaction between nitrite, sulfanilamide (SULFA), and N-naphthyl-ethylene-diamine (NEDA) to produce a pink AZO product which is spectrophotometrically measured. Briefly, 100 µL of deproteinized samples in duplicate was added to wells of a microtitre plate and mixed with 100 µL 0.8 % VCl${}_{3}$ in 1 M HCl, followed by addition of the Griess reagents (50 µL 2 % SULFA, and 50 µL 0.1 % NEDA). The reaction mixture was incubated for 30 min at 37 ${}^{\circ }$C, and the ODs were read at 540 nm against a blank in the microplate reader. The assay was calibrated with sodium nitrite (NaNO${}_{3}$), and the results are expressed in terms of NaNO${}_{3}$ equivalents (µM).

Myeloperoxidase (MPO) activity was measured according to Quade and Roth (1997). The method is based on MPO-H${}_{2}$O${}_{2}$ oxidation of 3,3${}^{\prime }$,5,5${}^{\prime }$-tetramethylbenzidine hydrochloride (TMB) as a sensitive peroxidase substrate. Briefly, 15 µL of samples in duplicate was diluted with 135 µL of Hanks balanced salt solution (HBSS) without Ca${}^{2+}$ or Mg${}^{2+}$ in wells of a microtitre plate. After that, 50 µL of freshly prepared substrate buffer (20 mM TMB and 5 mM H${}_{2}$O${}_{2}$) were added. The colour change reaction was stopped after 2 min by adding 50 µL of 4 M sulfuric acid (H${}_{2}$SO${}_{4}$), and the ODs were read at 450 nm against a blank in the a microplate reader (BioRad, mod. 550). Results are expressed as ODs.

Total antioxidant capacity (TAC) was determined using the ferric reducing ability of plasma (FRAP) assay, as indicated by Benzie and Strain (1996). The method is based on the principle of the reduction of the ferric-tripyridyltriazine complex to the ferrous form, which causes the development of an intense blue colour, measurable spectrophotometrically. Briefly, 300 mM sodium acetate buffer, pH 3.6, 10 mM tris(2-pyridyl)-s-triazine (TPTZ) in 40 mM HCl, and 20 mM iron(III) chloride hexahydrate were mixed in a volume ratio of 10:1:1 to generate FRAP freshly, daily prepared solution. Subsequently, 10 µL of samples in duplicate was added to 300 µL FRAP solution in wells of a microtitre plate, and the ODs of the reaction mixture were read at 600 nm after 5 min of incubation at 37 ${}^{\circ }$C in the microplate reader. The assay was calibrated with iron(II) sulfate heptahydrate (FeSO${}_{4}$⚫7H${}_{2}$O), and the results are expressed in terms of FeSO${}_{4}$⚫7H${}_{2}$O equivalents (µM).

Total antioxidant activity (TAA) was determined applying the improved ABTS radical cation decolorization assay, as suggested by Re et al. (1999). The method is based on the reduction of the coloured 2,2${}^{\prime }$-azino-bis(3-ethylbenzothiazoline-6-sulfonic acid) (ABTS${}^{⚫+}$) radical cation to a colourless reduced form by the antioxidants of samples. The colour reduction is then measured spectrophotometrically. Briefly, (ABTS${}^{⚫+}$) radical cation was generated by the reaction of 7 mM ABTS with 2.45 mM of potassium persulfate (K${}_{2}$S${}_{2}$O${}_{8}$). The reaction mixture was incubated in the dark for 16 h at room temperature. Working solutions of ABTS${}^{⚫+}$ were obtained by diluting ABTS${}^{⚫+}$ at the OD of 0.700±0.02 at 734 nm. Subsequently, 300 µL of the ABTS${}^{⚫+}$ solution was added to 3 µL of samples in duplicate in wells of a microtitre plate, and the ODs were read at 660 nm after 6 min of incubation at room temperature in the microplate reader. The assay was calibrated with Trolox, and the results are expressed in terms of Trolox equivalents (mM).

Free radical scavenging activity (FRSA) was analysed by the use of DPPH reduction assay based on the reduction of the purple DPPH${}^{⚫}$ to 1,1-diphenyl-2-picryl hydrazine, as described by Blois (1958). Briefly, 25 µL of samples was mixed with 475 µL of 10 mM phosphate-buffered saline (PBS), pH 7.4, and 500 µL of a 0.1 mM DPPH solution in absolute methanol. The mixture was kept for 30 min in darkness at ambient temperature before absorbance reading at 520 nm against a blank using a spectrophotometer (SmartSpec 3000 UV/Vis, Bio-Rad, Segrate, Italy). The absorbance of the sample was compared with that of a reference sample containing only PBS and DPPH solution. The percentage of decrease of DPPH bleeding was calculated applying the following equation: % of inhibition =[1-(As/A0)]×100, where As is the absorbance of sample and A0 is the absorbance of the DPPH solution.

Total thiol (sulfhydryl group, -SH) levels (TTLs) were measured as indicated by Hu (1994). Thiols interact with 5,5${}^{\prime }$-dithiobis-(2-nitrobenzoic acid) (DTNB) and form a highly coloured anion with a maximum peak at 412 nm ($\varepsilon_{412}=13\;600$ M${}^{-1}$ cm${}^{-1}$). Briefly, 50 µL of samples was mixed with 1 mL of Tris-EDTA buffer (0.25 M Tris base, 20 mM EDTA, pH 8.2), and the ODs at 412 nm were read against a blank in the spectrophotometer. Next, 20 µL of 10 mM DTNB in absolute methanol was added to the solutions. After 15 min at ambient temperature, the ODs were read again against DTNB blank. TTLs were calculated as indicated by Hu (1994), and the results were expressed in micromolars.

Ceruloplasmin (CP) was estimated from its oxidase activity by using o-dianisidine dihydrochloride (ODD) as the substrate, as described by Schosinsky et al. (1974). Briefly, serum samples in duplicate were incubated at 37 ${}^{\circ }$C in the presence of ODD 7.88 mM in acetate buffer 0.1 M, pH 5.0. The absorbance reflecting the intensity of the colour was measured against a blank at 540 nm after 5 and 15 min in the spectrophotometer using 9 M H${}_{2}$SO${}_{4}$ for stopping the enzyme reaction. The oxidase activity of ceruloplasmin, expressed in units per millilitre (U mL${}^{-1})$ in terms of consumed substrate, was calculated from the difference between the two absorbance data according to Schosinsky et al. (1974).

### 2.3 Statistical analysis

Analytical data, presented as means ± standard deviation (SD), are the averages of three analyses performed for each parameter. A one-sample Kolmogorov–Smirnov test was used to determine normal distribution of data (p>0.05). Basic descriptive statistics, including the measures of central tendency and dispersion, were calculated. Linear regression analyses were performed in order to verify any possible correlations among the assessed parameters, and a probability level of p<0.05 was considered significant. All statistical analyses were performed using SigmaPlot for Windows Version 11.0 statistical software (Systat Software Inc., San Jose, CA, USA).

## 3 Results

Basic descriptive statistics of the assessed parameters are presented in Table 1. The inter-individual coefficients of variation (CVs) of all the assessed parameters were substantial, except for TAA (6.65) and at least in part TAC (21.54), FRSA (20.69), and TTLs (18.42), and the data are strongly scattered around the mean, indicating that the changes in these assays are relatively large.

**Table 1:** Basic descriptive statistics of the assessed parameters in goat kid serum.

Parameter	Mean	SD	SE	95 % confidence interval	25th–75th percentile	CV (%)
ROMs	1.322	0.365	0.082	1.151–1.492	1.035–1.574	27.62
TOS	15.34	8.66	1.94	11.28–19.39	8.82–18.08	56.49
${\mathrm{NO}}_{x}$	190.77	76.31	17.06	155.06–226.49	137.78–247.59	39.99
MPO	0.108	0.064	0.014	0.078–0.138	0.064–0.139	58.91
TAC	306.01	65.90	14.74	275.17–336.86	252.92–334.21	21.54
TAA	1.328	0.088	0.020	1.287–1.369	1.273–1.375	6.65
FRSA	75.84	15.69	3.51	68.49–83.18	76.63–83.04	20.69
TTLs	176.94	32.60	7.29	161.68–192.20	160.14–188.40	18.42
CP	27.19	10.35	2.31	22.34–32.03	22.81–33.12	38.08

ROMs: reactive oxygen metabolites (t-BHP equivalents, mM); TOS: total oxidant status (t-BHP equivalents, µM); ${\mathrm{NO}}_{x}$: nitric oxide metabolites (NaNO${}_{3}$ equivalents, µM); MPO: myeloperoxidase (optical densities, 450 nm); TAC: total antioxidant capacity (FeSO${}_{4}$⚫7H${}_{2}$O equivalents, µM); TAA: total antioxidant capacity (Trolox equivalents, mM); FRSA: free radical scavenging activity inhibition, %; TTLs: total thiol levels (µM); CP: ceruloplasmin (oxidase activity, U mL${}^{-1})$; SD: standard deviation; SE: standard error; CV: coefficient of variation (inter-individual).

**Figure 1:**
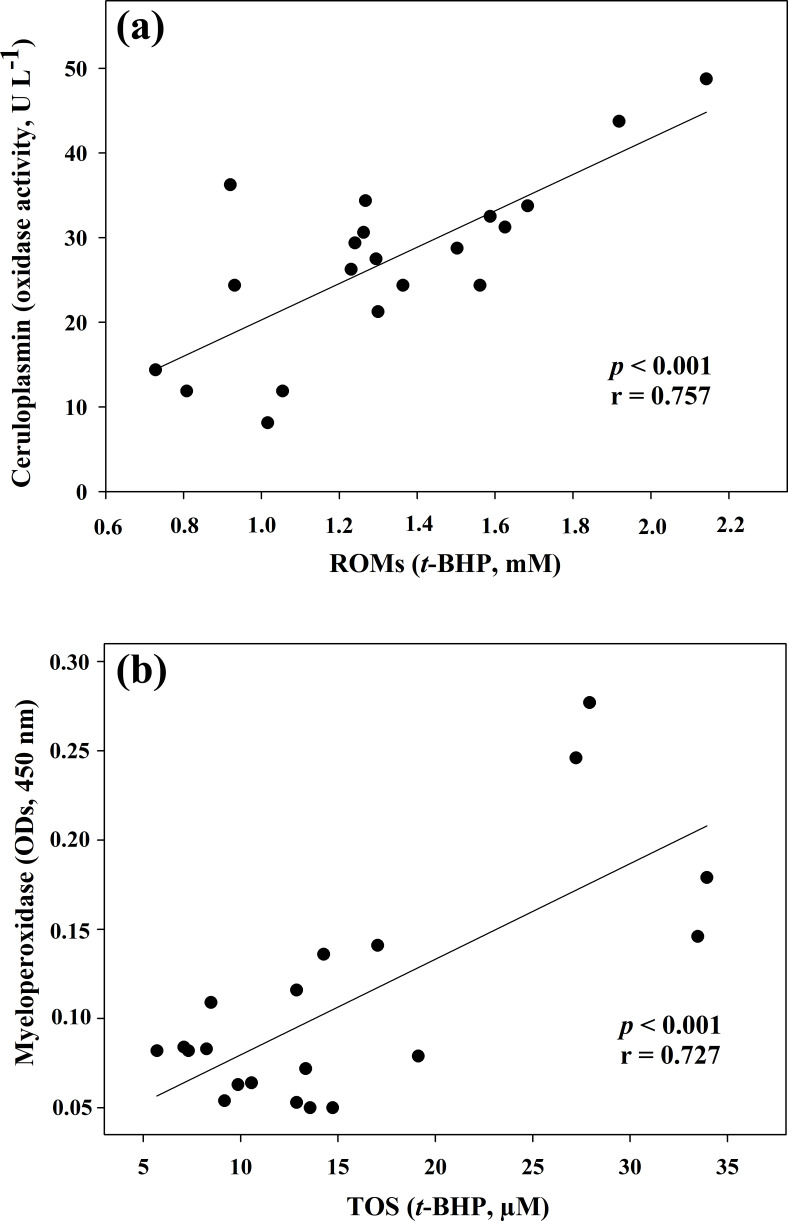
Regression analysis between ceruloplasmin and reactive oxygen metabolites (ROMs) **(a)** and between myeloperoxidase and total oxidant status (TOS) **(b)** in goat kid serum.

**Figure 2:**
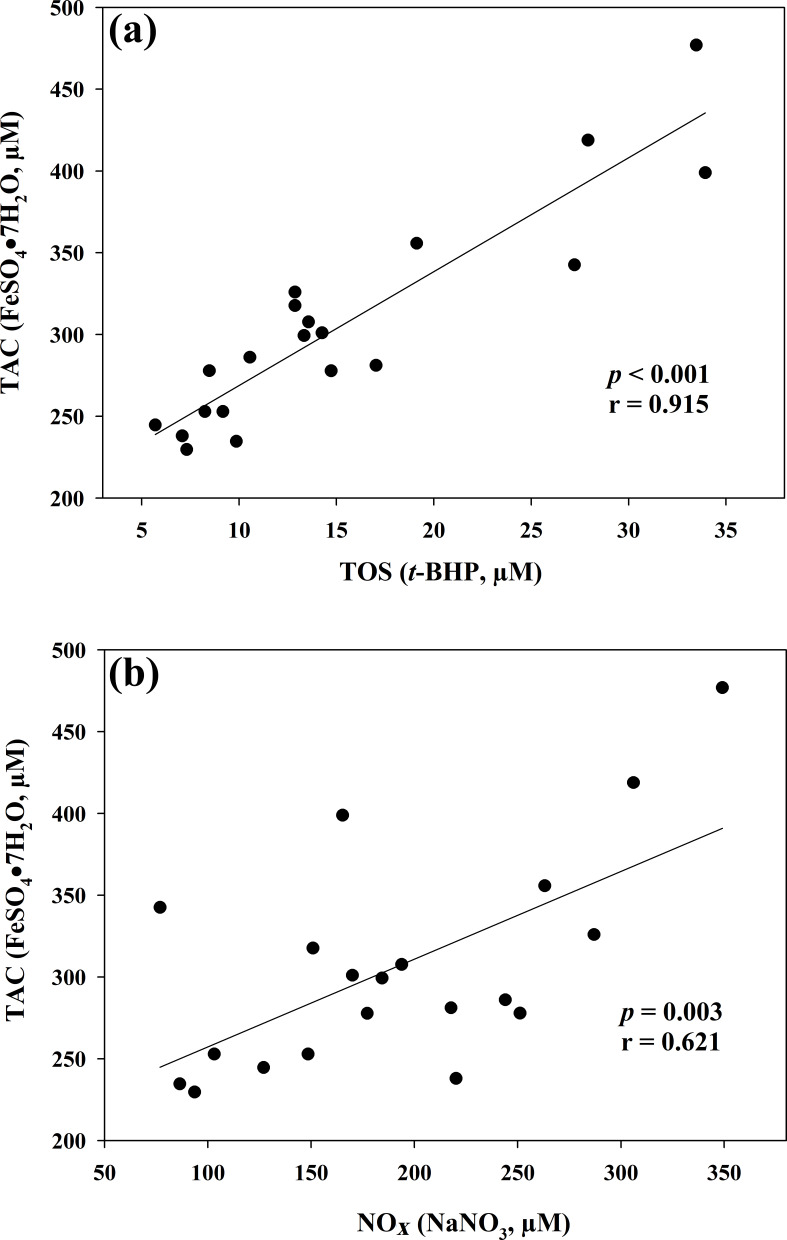
Regression analysis between total antioxidant capacity (TAC) and total oxidant status (TOS) **(a)** and between total antioxidant capacity (TAC) and nitric oxide metabolites (${\mathrm{NO}}_{x}$) **(b)** in goat kid serum.

**Figure 3:**
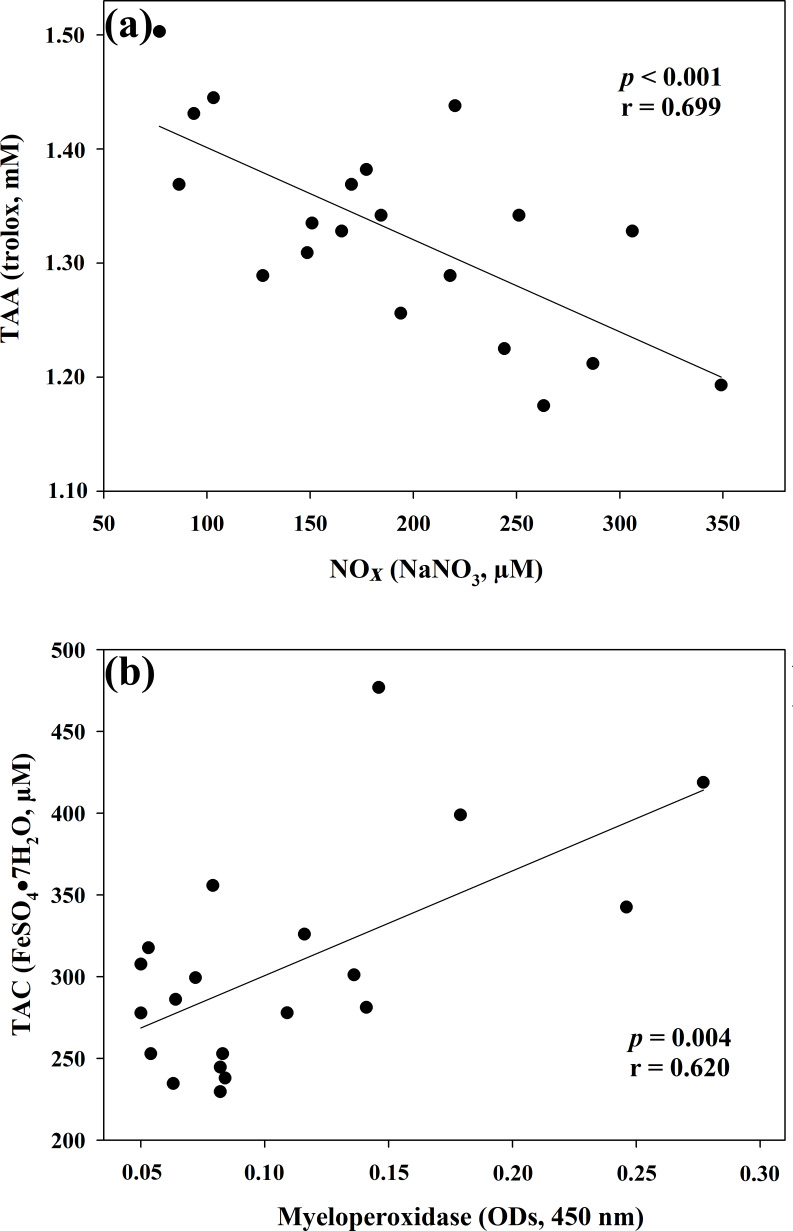
Regression analysis between total antioxidant activity (TAA) and nitric oxide metabolites (${\mathrm{NO}}_{x}$) **(a)** and between total antioxidant capacity (TAC) and myeloperoxidase **(b)** in goat kid serum.

The results of the comparisons among the evaluated parameters are presented in Table 2. When a correlation between some parameters was demonstrated, the values of the correlation coefficient and significance were high. As regards the two oxidant assays (ROMs and TOS), no correlation appeared (r=0.122), but ROMs were strongly correlated with CP oxidase activity (r=0.757, p<0.001, Fig. 1a), and TOS with MPO activity (r=0.727, p<0.001, Fig. 1b) and TAC (r=0.915, p<0.001, Fig. 2a). ${\mathrm{NO}}_{x}$ assay, as a marker of nitrosative stress, was correlated with TAC (r=0.621, p=0.003, Fig. 2b) and TAA (r=0.699, p<0.001, Fig. 3a). No correlations appeared among the assessed antioxidant assays, but TAC was correlated with MPO activity (r=0.620, p=0.004, Fig. 3b).

**Table 2:** Correlation matrix among the assessed parameters in goat kid serum.

	TOS	${\mathrm{NO}}_{x}$	MPO	TAC	TAA	FRSA	TTL	CP
ROMs	0.122	0.184	0.193	0.121	0.053	0.175	0.163	0.757${}^{***}$
TOS	–	0.370	0.727${}^{***}$	0.915${}^{***}$	0.180	0.436	0.160	0.106
${\mathrm{NO}}_{x}$	–	–	0.220	0.621${}^{**}$	0.699${}^{***}$	0.200	0.056	0.140
MPO	–	–	–	0.620${}^{**}$	0.178	0.188	0.044	0.034
TAC	–	–	–	–	0.424	0.249	0.081	0.084
TAA	–	–	–	–	–	0.047	0.047	0.159
FRSA	–	–	–	–	–	–	0.253	0.075
TTLs	–	–	–	–	–	–	–	0.210

ROMs, TOS, ${\mathrm{NO}}_{x}$, MPO, TAC, TAA, FRSA, TTLs, CP: see Table 1. ${}^{**,\;***}$ indicate a statistical significance for p<0.01 and p<0.001, respectively.

## 4 Discussion

In the present study different spectrophotometric methods to measure the redox balance in goat kid serum, together with MPO activity and CP oxidase activity, were applied. Regarding the analysed oxidant assays, data about ROMs and TOS were not correlated, as previously shown in human samples (Erel, 2005; Jansen and Ruskovska, 2013). Although both ROMs and TOS assays are based on iron-mediated mechanisms, the principles of detection are quite different, and this could be the reason for the lack of correlation (Erel, 2005; Jansen and Ruskovska, 2013). In reality, the ROM test, given by Alberti et al. (2000) as a specific measurement of hydroperoxide levels, was correlated neither with TOS nor MPO in the present results. In contrast, a highly significant correlation with CP oxidase activity, a glycoprotein known as a potent physiologic inhibitor of myeloperoxidase (Chapman et al., 2013), was evidenced in goat sera. It is worth noting that the correlation between ROMs and CP oxidase activity could be due to analytical interference. The ROM assay returns unrealistic hydroperoxide levels in some mammalian species, because CP and other serum components may interfere with the method (Erel, 2005; Kilk et al., 2014). This assumption was demonstrated in human and bovine sera by treating the samples with sodium azide, a strong inhibitor of CP (Erel, 2005; Kilk et al., 2014). Thus, all this may have caused an overestimation of ROM levels also in goat samples, and it could be responsible for the lack of correlation with TOS. Therefore, further studies are needed to understand the possible interference of goat serum CP in the ROM assay.

Contrary to what was observed in human medicine (Jansen and Ruskovska, 2013), in which no correlations appear between ROMs or TOS and antioxidant assays, TOS of goat serum was highly correlated with TAC, measured as FRAP. The lack of correlation between ROMs and TAC confirmed what was previously observed in small ruminants (Cecchini et al., 2018), unlike in other species (Fazio et al., 2015 and 2016). It is interesting to note the correlation between TOS and MPO activity, an oxidative neutrophil enzyme taking part in the anti-microbial system and inflammatory regulation. Its release during inflammation may lead to irreversible protein and lipid modification, increasing levels of lipid hydroperoxides (Nazligul et al., 2011), which are the main components detected by the TOS assay (Erel, 2005).

As regards the biomarker of nitrosative stress, ${\mathrm{NO}}_{x}$ levels, the stable end-products of nitric oxide radical (NO${}^{⚫}$), are strongly correlated with the antioxidant levels measured by a TAC assay, as previously shown in jumper horses (Fazio et al., 2016) and humans (Padhy et al., 2015). In humans, a correlation between TOS e ${\mathrm{NO}}_{x}$ levels was also shown (Padhy et al., 2015), whereas in our study the correlation was not observed (r=0.370; p=0.10). ${\mathrm{NO}}_{x}$ levels were also significantly correlated with TAA, as well as MPO activity with TAC, as previously shown in human disorders, in which the increased ${\mathrm{NO}}_{x}$ levels and MPO activity are suspected to be involved in OS (Akcay et al., 2012; Schuh et al., 2018).

Regarding the assessed antioxidant assays, no correlations were found. Unlike humans in which TAC and TAA are highly correlated (Jansen and Ruskovska, 2013), our results in goat kids showed correlation levels above the cut-off value (r=0.424, p=0.062). In humans, this correlation was related to the serum content of uric acid, one of the major components determining the antioxidant status in human serum (Erel, 2004; Jansen and Ruskovska, 2013), as previously in vitro observed by Benzie and Strain (1996) and Re et al. (1999). The lack of correlation between TAC and TAA in our data could be explained by the lower uric acid content in goat compared to humans (Silanikove et al., 1996). Thus, the assessed antioxidant assays were less influenced by the uric acid contribution, which represents 60 % of the antioxidant capacity measured by FRAP assay in human samples (Cao and Prior, 1998). Furthermore, the different technology on which the two assays are based inevitably leads to incomparable and therefore unrelated results. The technology of FRAP assay, based on the reduction of iron from ferric to ferrous form as a final indicator, does not allow the measurement of the SH group containing antioxidants, such as glutathione (GSH) and albumin, the latter responsible for approximately 30 % of the antioxidant capacity assessed by the ABTS${}^{⚫+}$ decolorization assay (Cao and Prior, 1998; Janaszewska and Bartosz, 2002). So, the contribution of SH group containing compounds in FRAP assay is very low (Cao and Prior, 1998), whereas uric acid, α-tocopherol, bilirubin, and ascorbic acid are the main contributors (Benzie and Strain, 1996).

TTLs are the antioxidant assay measuring the occurrence of thiol (-SH) groups in biological samples, mainly bound to proteins as sulfhydryl groups in a side chain of cysteine. Since thiol groups react similarly to GSH, their contribution to the FRAP assay is expected to be low (Janaszewska and Bartosz, 2002), as shown by the absence of correlation (r=0.084) obtained by the present data.

Very few studies were published regarding the correlation between TTLs and other antioxidant assays. In humans, a weak correlation between TTLs and antioxidant capacity, measured with an ABTS${}^{⚫+}$ decolorization assay, was shown (da Costa et al., 2006). The observation allowed the authors to suggest the use of the TTL assay as an auxiliary or substitute for the ABTS${}^{⚫+}$ decolorization assay when an evaluation of the total antioxidant status in human serum is required (da Costa et al., 2006), given that thiol protein groups represent about the 50 % of the total antioxidant power in healthy humans (Erel, 2004).

Except for TAA, the present results showed a great inter-individual variation for both oxidant and antioxidant assays, as previously shown in goats and other livestock. In these species analytical data are generally characterized by great individual variability (Cecchini et al., 2018 and 2019; Celi et al., 2010; Di Trana et al., 2015; Oikonomidis et al., 2017). This feature, a possible cause of inaccurate results, seems to be typical of some physiological studies in which livestock, usually selected for production traits and not for physiological responses, are involved. Besides, the results obtained with different assays can strongly differ depending on the method and technology of each assay, mainly based on the contribution of individual oxidant/antioxidant substances present in different samples.

Therefore, the use of different assays integrated into a panel of measurements should be strongly recommended to verify dutifully any changes in the redox balance, mainly in livestock in which reference values for each assay are lacking.

## 5 Conclusions

This study suggests that the evaluation of the redox potential should involve multiple assays due to the different analytical technologies on which their assessments are based. The simultaneous use of different analytical assays should be considered a key factor for the assessment of the redox balance in goat serum, to verify whether animals are experiencing OS or to evaluate the possible benefit from an antioxidant-enriched diet.

## Data Availability

The original data are available upon request to the corresponding author.
